# Cross-age effects on forensic face construction

**DOI:** 10.3389/fpsyg.2015.01237

**Published:** 2015-08-21

**Authors:** Cristina Fodarella, Charity Brown, Amy Lewis, Charlie D. Frowd

**Affiliations:** ^1^Department of Psychology, University of Winchester, Winchester, UK; ^2^School of Psychology, University of Leeds, Leeds, UK; ^3^The School of Psychology and Neuroscience, University of St Andrews, Fife, UK

**Keywords:** own-age bias, face perception, facial memory, facial composites, PRO-fit, glasgow face matching test

## Abstract

The own-age bias (OAB) refers to recognition memory being more accurate for people of our own age than other age groups (e.g., Wright and Stroud, 2002). This paper investigated whether the OAB effect is present during construction of human faces (also known as facial composites, often for forensic/police use). In doing so, it adds to our understanding of factors influencing both facial memory across the life span as well as performance of facial composites. Participant-witnesses were grouped into younger (19–35 years) and older (51–80 years) adults, and constructed a single composite from memory of an own- or cross-age target face using the feature-based composite system PRO-fit. They also completed the shortened version of the glasgow face matching test (GFMT; Burton et al., 2010). A separate group of participants who were familiar with the relevant identities attempted to name the resulting composites. Correct naming of the composites revealed the presence of an OAB for older adults, who constructed more-identifiable composites of own-age than cross-age faces. For younger adults, age of target face did not influence correct naming and their composites were named at the same level as those constructed by older adults for younger targets. Also, there was no reliable correlation between face perception ability and composite quality. Overall, correct naming was fairly good across the experiment, and indicated benefit for older witnesses for older targets. Results are discussed in terms of contemporary theories of OAB, and implications of the work for forensic practice.

## Introduction

Individuals can effortlessly and accurately detect the age of a face across their life span (e.g., Rhodes and Anastasi, 2012). Age-indicative information can influence face-recognition accuracy, and lead to an own-age bias (OAB) where facial memory is stronger for those of our own than other age groups (Wright and Stroud, 2002; for a review, see Wiese et al., 2013a). Findings for the OAB have been replicated across ages (Rhodes and Anastasi, 2012) and contexts, such as eyewitness line-up studies (Wright and Stroud, 2002) and old/new decision tasks (Wiese, 2012).

It is worth mentioning that the own-*race* bias (ORB) resembles a separate phenomenon, whereby individuals are better able to remember faces belonging to their own-race relative to another race (e.g., see Meissner and Brigham, 2001). This has led researchers when attempting to explain effects of OAB to draw upon accounts originally put forward for the ORB: The assumption is that both own-race and OAB are examples of a more general underlying phenomenon.

Several accounts have commonly proposed that a social categorization mechanism contributes to explaining group biases (e.g., Sporer, 1991; Levin, 2000). For example, Hugenberg et al.’s (2010) *categorization–individuation model* (*CIM*) theorizes that during the processing of a face, individuals engage either in categorization or individuation. Categorization leads to faces being encoded in terms of the social category to which they belong. This is thought to hinder the ability to discriminate between faces during recognition. Conversely, individuation leads to faces being encoded with regard to individualistic characteristics which would promote later recognition. In terms of the OAB, cross-age faces may be perceived with regard to the age category to which they belong (categorization), whilst own-age faces may be perceived with more unique and individuating information (individuation). The impact of this effect results in the superior recognition of own-age faces.

The configural-feature hypothesis may also apply. This account proposes that highly-familiar faces, identities which are encountered frequently, are recognized based to a greater extent upon the configural information they contain (i.e., via the encoding of spatial relationships between some or all facial features; see Peterson and Rhodes, 2003) compared to information about individual facial features (eyes, nose, mouth, etc.) Facial memory is generally stronger when faces are perceived configurally. Therefore, own-age faces may be processed configurally and thereby more-effectively, whilst cross-age faces may be processed featurally, leading to an OAB (see Rossion and Michel, 2011, for a review). However, research comparing younger and older adults on holistic/configural processing of young and old faces is sparse. Wiese et al. (2013a) examined this issue using the well-known composite-face effect as an indicator of holistic processing. They found both younger and older adults were better at the task of discriminating two face halves when presented as misaligned compared to aligned, and this effect was particularly marked for young relative to old faces. This finding indicates that the effective application of holistic processing was determined by the age of the target face *per se*, rather than any effects of own-group bias. Nevertheless, using ERP measures, Wiese et al. (2013b) did observe an own-age advantage for younger, but not older participants when examining N250r, a component interpreted as reflecting enhanced access to face representations. More generally, there was evidence that holistic processing by older compared to younger adults was overall less efficient.

Further, it may be the case that increased contact with so-called “out” group members enables development of experience and expertise, both of which improve the ability to extract relevant information to aid recognition of out-group individuals (Meissner and Brigham, 2001). Normal ageing causes individuals throughout their life span to progress from one age group (e.g., younger adults) to another (e.g., older adults), and through the course of this process, older adults are likely to have gained considerable experience with faces of different age groups. However, it is debated whether accumulated contact over the lifespan influences the OAB, or recent daily-life contact only. There is evidence to support both views. For the former, there are many studies that show an OAB for younger but not older adults (e.g., Rhodes et al., 2008; Wiese et al., 2008; Havard and Memon, 2009). It could be theorized that this would be due to a difference in general experience with cross-age groups. Younger adults in general may not have had sufficient contact over their lifespan with older adults, leading to an OAB. In contrast, older adults must have had contact with all other age groups at some point during their lifetime, as they progressed through different age stages. Therefore, older adults have prior experience as a member of both age groups, leading to a lack of an OAB (see Wiese et al., 2008).

For the latter view, it is proposed instead that recent daily-life contact determines whether or not an OAB occurs. In support of this proposal, the OAB was not apparent when testing face recognition in young adult geriatric nurses (i.e., a job involving substantial contact with older adults) relative to young adult controls who as a group reported having low contact with an older adult population (Wiese et al., 2013c). Similarly, an OAB effect in older adults has also been shown to be mediated by different levels of contact. Wiese et al. (2012) included older adults who reported having either a high or a low level of recent contact with own-aged individuals relative to younger ones. Superior recognition of own-age faces (cf. cross-age faces) was apparent in those older adults reporting a high level of contact with an older adult population. In contrast, those with a more balanced contact to both younger and older adults did not show such bias. These findings indicate that previous experience of having been a member of the younger age group was not sufficient contact to diminish the OAB in *all* older adults, thereby suggesting an influential role for recent contact. The recent-contact hypothesis is further supported by a meta-analysis (Rhodes and Anastasi, 2012).

This opens up the question as to why some previous studies have not observed an OAB in older adults (e.g., Rhodes et al., 2008; Wiese et al., 2008; Havard and Memon, 2009). This null effect may be due to older adults tending to process the face featurally (through processing of individual face features) rather than configurally (processing an object as a whole; Murray et al., 2010). There may also be an associated age-related decline in the processing of facial information, one which causes older adults to perform worse (cf. young adults) when detecting, remembering and recognizing faces (for a review, see Ruffman et al., 2008). The configural-feature hypothesis proposes that familiar stimuli are processed more configurally rather than featurally; here, configural processing strengthens face-recognition memory to a greater extent than featural (Wiese et al., 2013b). Overall, a featural style of processing may hinder older adults from benefitting from improved recognition afforded by a greater reliance on configural information.

In an applied setting, identifying factors which influence face memory is important within a legal system. For example, eyewitnesses (witnesses and victims) are asked by police to construct a picture of the person they have seen commit a crime, an image known as a facial composite, and/or to identify a potential suspect from a police line-up (identity parade). Both of these forensic tasks involve face processing to a great extent and so may be susceptible to an OAB (e.g., Wright and Stroud, 2002). Here, our focus is on the former activity, people’s ability to construct identifiable facial composites. Composites provide evidence usually gathered in the early stages of a police investigation that can be crucial to locate potential suspects (see Frowd, 2015, for a general review of composites). They are usually constructed 2–3 days after the crime occurred, but are occasionally created on the same day. An OAB could occur here and, if so, could manifest itself in composites of own-age groups being more effective than those of cross-age groups. To date, no published research has formally explored this issue, and the current paper aims to plug this gap by including both a younger- and older-adult sample who construct composites of same- and cross-age faces.

When including an older-adult sample, age-related memory decline may be relevant. Facial-composite construction using traditional “feature” systems involves detailed recall of facial features from memory (normally using cognitive-type interviewing procedures). Unfamiliar-face recognition is also involved as eyewitnesses are required to select individual facial features (e.g., eyes, nose, mouth) to build a face. Therefore, as both face recall (Wright and Holliday, 2007) and face recognition (Bartlett and Fulton, 1991) are impaired with advancing age, composites produced by older adults may be less effective than those produced by younger adults. Nevertheless, Komes et al. (2014) found that despite dividing their older adult sample into those exhibiting low versus high face recognition memory performance, both groups showed an equivalent bias toward recognizing own- versus other-race faces (i.e., an own-race bias). This suggests that even when memory is less effective individuals may still display a memory bias toward own-group faces, in this case, own-race faces. Similarly, in the present study we may find an OAB emerges over and above any more general age-related memory decline that becomes apparent in the task at hand. In this regard, it is worth noting that one study in the composite area (Frowd et al., 2005a) included older adults in their sample, but found no reliable relationship between age of face constructor and identification of resulting composite; however, while age of target varied considerably across the stimuli set, this property was a random variable and so the design may not have been sufficiently powerful to be able to detect an OAB, should one exist. The aim of the current study, therefore, is to formally assess whether age-related differences exist for composite-face production.

In summary, we investigated whether an OAB effect occurs during composite construction for both younger and older participants. These participant “witnesses” (face constructors) were grouped into younger and older adults and constructed a single feature-based composite of either an own- or cross-age target face. As there are fairly-large individual differences in ability to construct faces from memory (e.g., Frowd et al., 2016), and face recognition is an important component for composite construction (e.g., Frowd et al., 2008), participant-witnesses also completed the shortened Glasgow Face Matching Test (GFMT; Burton et al., 2010), to initially investigate whether a relationship exists between face-perception ability and composite quality.

On the basis of the aforementioned face-recognition research, it was expected that an OAB effect would occur when constructing composites, and that this effect would be stronger for a younger than an older adult group. Also, given evidence of age-related decline in both recall and recognition, older (cf. younger) adults were expected to produce less effective composites in general.

## Experiment

To mirror real-life criminal investigations, participants who constructed the composites were required to be unfamiliar with the target faces, whilst participants who later attempted to identify the composites were required to be familiar with these identities. To satisfy this constraint on familiarity, previous research concerning composite construction has made use of target categories such as international sports players (e.g., snooker or football players; e.g., Frowd et al., 2007, 2010). This enables the recruitment of people who are not fans of the sport and hence unfamiliar with the target identities, for face construction and subsequently recruitment of sports fans (familiar with the target identities) for naming the resulting composites. Here premiership footballers and international/premiership football managers were used on the basis that these two groups contain individuals that fall into two separate age groups (younger, 22–33 years; older, 49–72 years) allowing age of target to be treated as a categorical variable. This allowed recruitment of participants (constructors) who were unfamiliar with the targets—people who where non-football fans—to create two groups that were mutually exclusive by age (younger, 19–35 years; or older, 51–80 years). These participants made a single composite of an unfamiliar target identity belonging either to their own or the other-age category. Subsequently, football fans were recruited as composite-namers who were familiar to the targets. Football fans are likely to know both footballer players and managers which allowed us to adopt a more powerful within-participants design for composite naming: all naming participants attempted to name both the younger (football players) and older (football managers) target identities. The two stages of composite construction and naming required a quite different design and procedure, as described below. The research was approved by the School of Psychology Ethics’ Committee at the University of Leeds and complied with the relevant regulatory standards.

### Composite-construction Stage

#### Design

Participants (“constructors”) encoded a target face and then created a single composite from memory using the PRO-fit “feature” system in current police use (Frowd, 2015). This single-session design produces more-identifiable composites than designs involving a long retention interval (e.g., of 1 or 2 days) between target encoding and face production (Frowd et al., 2005b) and should improve the chances of observing an OAB, should one exist.

The two factors of target age and constructor age were each treated as categorical variables and implemented at two levels, “older” and “younger”; age groups in both cases were mutually exclusive. The targets were premiership footballers (“younger” targets) and premiership and international football managers (“older” targets). Thus, a 2 (constructor age: younger vs. older) × 2 (target age: younger vs. older) between-participants design was employed. The experimenter was unaware of the identities of the target photographs and the target-age conditions to which participants had been randomly allocated.

#### Materials

The targets were front-facing color photographs located via an internet search: 10 white male premiership-level football players (age: 22–33 years; *M* = 28.0; SD = 4.0 years) and 10 international/premiership-level football managers (age: 49–72 years; *M* = 60.5; SD = 8.5 years). No one wore particularly distinctive features such as glasses, beard or jewelry. The size of each target image was approximately 6 cm (wide) by 8 cm (high). Each target was printed twice on single sheets of A4 paper in color, producing 40 targets in total: (i) 10 younger targets for younger participants, (ii) 10 younger targets for older participant, (iii) 10 older targets for younger participants and (iv) 10 older targets for older participants. Each target picture was shown to a different constructor to build a composite of that target face.

The shortened version of the Glasgow Face Matching Task (GFMT), a measure of individual face-processing ability, was administered to all constructors. Participants were presented with 40 pairs of faces, and asked to make same/different judgments. Scores were calculated out of 40, with one point awarded for each correct detection or discrimination.

Older adult participants were additionally screened using the Montreal Cognitive Assessment Tool (MoCA; Nasreddine et al., 2005). This cognitive-screening tool takes little time to administer and assesses potential mild cognitive impairment. Cognitively-intact older adults typically score in the range of 26 or above. Therefore, adults scoring 26 or less on this assessment did not participate to completion in the study. This was to ensure that any effects of age on composite construction quality were not masked by the presence of abnormal cognitive decline within our older adult sample. PRO-fit software version 3.5 was used to construct the composites.

#### Participants

The two age categories of participants for face construction were selected to be close to those of the younger and older target stimuli (see Materials and Methods). They were mutually-exclusive and were in keeping with age categories used within previous OAB research. For example, within their meta-analysis Rhodes and Anastasi (2012) grouped participants aged 18–35 within a single “young” age group. Further, whilst previous research by Wolff et al. (2012) did split their participants into a “young” age group (19–29 years) and a “young middle” age group (30–44 years), they found comparable memory across these two groups for faces ranging from 18 to 44 years, with both age groups showing less accurate memory for older faces relative to young and young middle-aged faces. Similarly, our younger composite constructor group consisted of individuals spanning 19–35 years. Some researchers have also included a wide variety of ages within their older adult samples (e.g., >55 years, Rhodes and Anastasi, 2012; 63–92 years, He et al., 2011; 55–89 years, Anastasi and Rhodes, 2005; 64–86 years, Wilcock et al., 2007). However, some have distinguished between old (>75 years) and young-older adult participants (55–74), and there is evidence that old compared to young-older adults may perform differently on some memory tasks, including those that involve event recall (e.g., Wright and Holliday, 2007) or recognition of faces of different ages (e.g., Bäckman, 1991). In the present experiment, the older adult sample consisted of individuals aged 51 to 80 years, but included predominantly young-older adults (17 out of 20 participants were aged below 65). Whilst one may be inclined to suggest that the age difference between our younger (19–35) and older (51–80) age categories is small, Wolff et al. (2012) showed that there is indeed a performance difference between these age groups (19–44 vs. > 45 years) with regards to OAB.

The younger adult group (*N* = 20) was recruited from the University of Leeds via opportunity sampling. The age range is 19 to 35 years (*M* = 24.6; SD = 4.0, Kurtosis = 1.61; Skew = 1.28). The older adult group (*N* = 20) was recruited through word of mouth (in the Leeds area, North East England). Their age ranged from 51 to 80 years (*M* = 60.7; SD = 7.6; Kurtosis = 1.94; Skew = 1.39).

Participants were advertised on the basis of being unfamiliar with international footballer players and managers, spoke English as their first language and did not have regular recent daily-life contact with people of a different age group other than themselves. A high level of recent contact with people from the other-age group has previously been associated with a reduction in the OAB in recognition measures (Wiese et al., 2012, 2013a), and may mitigate against our observing any reduced effectiveness associated with constructing composites of other-age faces (Wiese et al., 2012, 2013a). We therefore asked participants whether they had regular contact, such as in an occupational capacity, with people from the other age category within the last 5 years. No participant constructing a composite of a target from the other-age category reported having pronounced job-related or other types of contact with people of the other-age group. All participant-constructors reported having normal or normal-to-corrected vision. Participants were paid £5 for their time.

#### Procedure

Participants were tested individually throughout by the researcher. After giving informed consent, the older adult group completed the MoCA at least 30 mins prior to the experiment. Participants next attended a testing session lasting from 45 to 60 mins. They were told that they would create a composite of an unfamiliar target face. Participants then removed a target picture from an envelope (randomly selected by target and by condition, without replacement) and reported whether it was a known identity. If it was familiar, they were asked to select another at random. For the first face that was reported to be unknown, participants were given 60 s to remember it. One person reported that all available targets were familiar and was replaced by another person, to give the sample described in Participants. Following this procedure aimed to ensure that all participants constructed a composite of an unfamiliar face, as would be the norm for real witnesses.

The remaining procedure was self-paced. An Enhanced Cognitive Interview, as described by Fodarella et al. (2016), was administrated to allow participants to recall detailed information about the appearance of their target face; this interview was initiated with rapport-building, and was followed by context reinstatement, free recall and cued recall. Constructors were also told that it was acceptable to state if they did not recall specific features during cued recall; this instruction was important to state as research suggests that older adults have a tendency to guess during recall tasks (Huff et al., 2011). The researcher operated the PRO-fit composite software to allow participants to construct a single composite of the target from memory. The procedure used to construct the composites is fairly detailed, and is described in full in Fodarella et al. (2016); in brief, participants selected individual features to match their description of the face, with each selected feature resized and positioned on the face with the aim of achieving the best likeness possible. Finally, participants completed the 40-item GFMT, and were debriefed.

### Composite-naming Stage

#### Design

A separate group of participants were invited to name the composites, to give a method of assessing the effectiveness of composites which is similar to their use forensically (e.g., Frowd et al., 2005a). Participants were recruited on the basis of their reported familiarity with both footballers who play within the premiership in the UK and those individuals who manage international and premiership football teams. The design was 2 (between-participants: constructor age) × 2 (within-participants: target age) Mixed-Factorial. The 40 composites constructed in the previous stage of the experiment were separated into two equal sets by categorical age of constructor. Composites of two young-male and two older-male unfamiliar targets (so-called “foils”) were added to each set; these additional composites were of unfamiliar identities in general (and not of football players or managers) and were included to limit naming by guessing and to increase ecological validity (e.g., Frowd et al., 2016).

The number of participants required in the naming stage was chosen to be able to detect a small effect size when their data were subject to a regression type analysis. This was based on a G*Power analysis (version 3.9.1.2; Faul et al., 2009) with a small effect size (Odds Ratio *OR* = 1.5). Alpha was set at, *a* = 0.05, and power, 1–β = 0.95, with an equal number of participants viewing composites belonging to each constructor age group (Younger vs. Older adults; the between-participants factor). We assumed that at the very least a small amount of variance associated with age of constructor would be explained by the presence of the within participants factor, age of target, and therefore estimated a squared multiple correlation co-efficient of *R*^2^ = 0.1 as an additional input parameter. A normal distribution was assumed for each predictor. Based on these relatively conservative parameters the analysis indicated that about 10 participants per group would be sufficient. We exceeded this lower estimate by recruiting 16 participants in each group (total *N* = 32).

#### Materials

**FIGURE 1:**
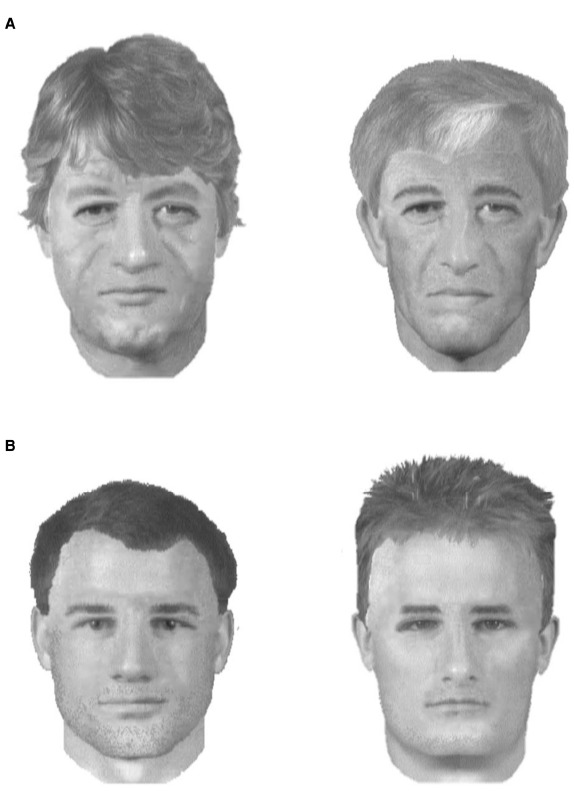
**Example composites constructed of the professional footballer manager Arsene Wenger, an older age target (A) and the professional football player John Terry, a younger age target (B).** The composites constructed by the younger age group are on the left, and those constructed by the older age group are on the right. Due to reasons of copyright, original pictures cannot be shown here.

The 40 actual composites and the four foil composites were proportionally sized to 15 cm (high) by 10 cm (wide) and printed in greyscale (the image mode of the composite system) on A4 paper. Figure 1 below shows example items across conditions. The 20 color target photographs from the construction stage were also required.

#### Participants

Thirty-two participants (1 female) were recruited via opportunity sampling in a local sports centre in the North East of England. Their age ranged from 21 to 59 (*M* = 30.8, SD = 10.2) years. Participants were assigned equally to the between-participants factor of constructor age. Each person was paid £2 for their time.

The majority of participants within the sample are male. While it is tempting to suppose that such a gender bias might skew results, previous research suggests that target gender does not strongly influence face recognition (e.g., Shapiro and Penrod, 1986) and, more specifically, gender has not been found to influence composite naming (Frowd et al., 2005b).

#### Procedure

Participants were tested individually. They were told that they would be shown a set of 24 composites to name, some of which were of premiership footballers or international or premiership football managers. It was also mentioned that some composites were of unfamiliar identities, to make the task more realistic. The relevant set of composites was then presented sequentially for participants to name, randomly assigned to constructor age with equal sampling. Next, the 20 target photographs were presented likewise for naming. This acted as a familiarity check to ensure participants were familiar with the majority of the target identities. According to an *a priori* rule, participants’ data were excluded if less than 16 targets were named correctly. This situation occurred on five occasions. Data from these participants were not included in the analysis, and further participants were recruited as replacements in the relevant conditions (to give the sample described above). The naming task was completed in about 15 mins per person, including debrief.

## Results

### Spontaneous Naming

Participant responses to composites were checked for missing data (of which no cases were observed) and scored for accuracy: a numeric value of 1 was assigned when the correct name was given and 0 otherwise. Overall, 52 responses were correct out of a possible 640. Responses to target photographs were handled in the same way, and 603 were correct. Target naming was thus considerably higher than composite naming, but this is the usual situation as composites are prone to error and are rarely named perfectly. However, failure to recognize a target does suggest that its corresponding composite could not be recognized either, and so, for each of these cases, the relevant composite was scored as missing data (i.e., not included in the subsequent analysis).

Composite-naming scores were subjected to Logistic Regression for age of target (younger vs. older adult) and age of constructor (younger vs. older adult). A full-factorial model was built and each predictor was subject to sequential removal (for *p* > 0.1) using Backward LR: age of constructor was removed in Step 1 (*p* = 0.61). The resulting model was reliable [*X*^2^(2) = 9.4, *p* = 0.009, *R*^2^(Cox and Snell) = 0.015, *R*^2^(Nagelkerke) = 0.035] with a good fit (Hosmer and Lemeshow, *p* = 0.88).

Age of target was reliable [regression coefficient *B* = 0.6, SE*(B)* = 0.3, *p* = 0.039], with an advantage for older over younger targets [*Exp(B)* = 1.9]^1^. This predictor was qualified by age of constructor [*B* = 1.4, SE*(B)* = 0.6, *p* = 0.025, *Exp(B)* = 3.9] (Table 1) since (using two-tailed Fisher Tests) the advantage of target age was restricted to older constructors (*p* < 0.01, Odds Ratio *OR* = 3.4); also, for older targets, there was an advantage of older over younger constructors (*p* < 0.05, *OR* = 2.2).

**TABLE 1:** **The advantage of older constructors creating composites of older target faces**.

**Target**	**Constructor**	
	**Younger**	**Older**
Younger	7.6	4.9^a^
	(12/157)	(7/144)
Older	7.2^b^	14.8^a,b^
	(11/153)	(22/149)

Figures are percentage-correct accuracy calculated from responses in parentheses: summed correct responses (numerator) and total (correct and incorrect) responses (denominator). These data are for composites for which participants correctly named the relevant target (N = 603 out of 640). The model converged with significant predictors for age of target (p < 0.05), interaction (p < 0.05) and the Coefficient [B = –2.5, SE(B) = 0.2, p < 0.001, Exp(B) = 0.1]. See text for more details. ^a^p < 0.01; ^b^p < 0.05.

Participant responses to composites were also analyzed for mistaken names given, to provide an indication of willingness to offer any name (i.e., a guess); it is analogous to response *bias* in signal detection paradigms. After discounting correct responses to composites (*N* = 52) and screening out unfamiliar targets (*N* = 37), mean incorrect names were fairly frequent overall (*N* = 244/551, *M* = 44.3%)—a usual situation with composites (e.g., Frowd et al., 2016). Logistic Regression revealed that neither of the predictors (*p*s > 0.4) nor their interaction (*p* = 0.9) exerted a reliable influence on this DV.

### 40-item Glasgow Face Matching Task

A two-tailed *t*-test was run to compare scores on the GFMT across age groups. Previous findings indicate that there are no reliable age differences in task performance (Burton et al., 2010). Our findings replicate this null effect, *t* (38) = 1.1, *p* = 0.28.

As the GFMT is a measure of face-perception ability, it follows that those who are better at perceiving faces should also be better at constructing faces, as the latter should involve face perception. A one-tailed correlation between the correct-naming score for each composite and the relevant participant’s GFMT score was not significant, *r* (38) = 0.18, *p* = 0.13.

## Discussion

The current study aimed to investigate whether an OAB effect occurs in facial-composite construction. Older and younger adults viewed an own- or cross-age target face, and created a single composite from memory. The resulting composites were then given to further participants to name. Results of correct names given partially supported one of the hypotheses: OAB was found for older constructors, but—against predictions—not for younger adults.

Own-age bias refers to facial-recognition memory being more accurate for those of our own than cross-age groups (Wright and Stroud, 2002). The literature reveals somewhat inconsistent findings. Some studies indicate that an OAB occurs for all age groups across life span, that is, for both younger and older adults (e.g., Rhodes and Anastasi, 2012). Other studies find that it would only occur for younger adults, with no effect on older adults (Havard and Memon, 2009). The latter findings can be explained by the contact hypothesis which predicts that face recognition of other-age faces improves as a function of general contact with other-age faces that is accumulated throughout the lifespan. The former findings, however, are in line with a recent-contact hypothesis which indicates instead that it is recent daily-life contact with other-age faces (rather than contact gathered over the life span) that plays a role in mitigating OAB effects in face recognition (Wiese et al., 2012). The current study does not seem to fit with either hypothesis, with correct naming of composites suggesting an OAB for older but not younger adults.

The lack of OAB in younger adults is surprising given that previous research has consistently outlined the OAB effect in young adults (e.g., Wright and Stroud, 2002; Wiese, 2012). One possible explanation may be the fact that PRO-fit composite construction and face recognition rely upon different types of information—that is, featural versus configural information. PRO-fit being a feature system is likely to have led to featural processing, whilst limiting the ability to engage in configural processing during composite construction. In contrast, OAB may plausibly arise due to differences in the application of configural processing for own versus other age faces (e.g., Rossion and Michel, 2011). This may explain why younger adults showed no OAB. As configural processing is not suited to the feature-based PRO-fit task, younger adults did not benefit from having increased expertise and increased sensitivity to configural information for own compared to cross-age faces. Indeed recent evidence from work using ERP measures suggests younger compared to older adults process holistic information from faces more efficiently (Wiese et al., 2013a). Further, research stresses the importance of transfer-appropriate processing, a match between encoding and retrieval processes, to enable successful task performance (McBride and Abney, 2012). It would seem that face construction using a traditional feature system may not be capitalizing on configural information; in fact, participants who may be less efficient at processing faces holistically, and may therefore rely more on featural information (as in the older adults with older target faces), appear to benefit in this face-perception task. One way to investigate this account further would be to replicate the current research using a holistic composite system such as EvoFIT (e.g., Frowd et al., 2010). This type of system requires constructors to repeatedly select from arrays of complete faces, rather than by selecting individual features, with the aim of maximizing construction of configural cues. It does seem to be the case that this system is a more effective method of accessing memory since mean naming of its composites has been reported to be around 50% correct following a 24 h retention interval (e.g., Frowd et al., 2013, 2016). If the above proposed account is correct, an OAB would be expected to occur for younger adults. This could be due to the holistic system enabling younger adults to use their expertise, leading to an OAB. In contrast, an OAB may now not occur in older adults.

So, our data indicates an OAB in older adults, which is in line with some research showing an OAB for this age group (Rhodes and Anastasi, 2012). As the ability to engage in configural processing declines with age, older adults may therefore engage more in featural processing as a consequence (Murray et al., 2010). This is likely to have been suited to the feature-based PRO-fit task, thereby enabling an OAB for older adults but not younger ones. However, this cannot be the only explanation, as the use of feature-processing *per se* would have led to better-quality composites for both own- and cross-age faces in older adults.

Hugenberg et al.’s (2010) *CIM* may aid in explaining the effect further. The CIM proposes that individuating information about a face is encoded for own-group faces, thereby facilitating memory. Taking this into account, it may be that older adults were able to extract feature-based individuating information from own-age faces, which may have aided construction of good-quality own-age composites.

Taking into account age-related memory decline (Havard and Memon, 2009), and the fact that older eyewitness memory recall is less detailed and accurate (Wright and Holliday, 2007), it was hypothesized that older adults may produce less-identifiable composites than younger adults. However, no difference was found, and this is consistent with past research in the composite area (Frowd et al., 2005a). In fact, our data indicates a situation in which older adults actually outperformed younger adults. However, within the current study the older adult sample predominantly consisted of adults aged 51–65 years (17 out of 20 participant-constructors). Therefore, as memory declines steadily with age (Grady and Craik, 2000), it may be that age-related memory decline was not strong enough to be observed within our older-adult sample. Replicating this research with an older sample of a smaller age range (70–80 years) would be beneficial to firm-up conclusions.

Our findings also indicated no difference across the two age groups in face perception ability as measured by the 40-item GFMT measure. This suggests that the ability to detect similarities/differences in faces does not decline with age. This is in line with previous research (Burton et al., 2010). However, we expected to find that those scoring high on the GFMT would produce more-identifiable composites, as face construction requires the ability to process faces. No significant positive correlation was found between composite quality and GFMT score. However, future research could also consider incorporating alternative individual difference measures. For example, recognition memory likely plays a role in face construction, and assessing the relationship between measures targeting face recognition memory ability and face construction would aid understanding of the extent to which face construction relies upon an individual’s ability to effectively utilize information residing in memory (e.g., memory for configural versus feature information).

With regard to a real-life application, the data suggest a lack of age difference for constructors—that is, older eyewitnesses produce composites to a similar quality to those of younger witnesses. Identification of composites is likely to be better when older adults construct faces of a similar age to themselves, outperforming younger adults. Thereby, composites are likely to be more effective from an elderly witness (cf. younger witness) when the offender is also elderly. This, as we have argued, may differ depending on which composite system is used.

In summary, the current paper is the first to formally investigate whether an OAB occurs during composite construction. Findings indicate that an OAB occurred for older adults only. The mechanism for this OAB in older adults may simply be that these participants are better able to extract individuating feature-based information from targets of their own age, information which is beneficial for face production using the feature-based PRO-fit system.

### Conflict of Interest Statement

The authors declare that the research was conducted in the absence of any commercial or financial relationships that could be construed as a potential conflict of interest.

## Footnotes

1 For readers unfamiliar with the *Exp(B)* measure of effect size, it is equivalent to the Odds Ratio (OR)—the number of times one condition is more effective than another.
